# View the label before you view the movie: A field experiment into the impact of Portion size and Guideline Daily Amounts labelling on soft drinks in cinemas

**DOI:** 10.1186/1471-2458-11-438

**Published:** 2011-06-06

**Authors:** Willemijn M Vermeer, Ingrid HM Steenhuis, Franca H Leeuwis, Arjan ER Bos, Michiel de Boer, Jacob C Seidell

**Affiliations:** 1 Department of Health Sciences and the EMGO Institute for Health and Care Research, VU University Amsterdam, the Netherlands; 2 Faculty of Psychology and Neuroscience, Maastricht University, the Netherlands

**Keywords:** Portion sizes, Food labelling, Obesity prevention, Environmental interventions

## Abstract

**Background:**

Large soft drink sizes increase consumption, and thereby contribute to obesity. Portion size labelling may help consumers to select more appropriate food portions. This study aimed to assess the effectiveness of portion size and caloric Guidelines for Daily Amounts (GDA) labelling on consumers' portion size choices and consumption of regular soft drinks.

**Methods:**

A field experiment that took place on two subsequent evenings in a Dutch cinema. Participants (n = 101) were asked to select one of five different portion sizes of a soft drink. Consumers were provided with either portion size and caloric GDA labelling (experimental condition) or with millilitre information (control condition).

**Results:**

Labelling neither stimulated participants to choose small portion sizes (*OR *= .75, *p *= .61, CI: .25 - 2.25), nor did labelling dissuade participants to choose large portion sizes (*OR *= .51, *p *= .36, CI: .12 - 2.15).

**Conclusions:**

Portion size and caloric GDA labelling were found to have no effect on soft drink intake. Further research among a larger group of participants combined with pricing strategies is required. The results of this study are relevant for the current public health debate on food labelling.

## Background

The mean portion size of soft drinks has increased in the past decades [1,2], and over time larger portion sizes have been added to the product lines [3,4]. Soft drinks have been recognized as potentially important contributors to obesity [5] and it has been demonstrated that serving larger soft drink portions results in increased beverage consumption [6]. Next to the availability of larger portion sizes, 'portion distortion' [7,8] might stimulate the consumption of increasingly larger amounts of soft drinks.

Nutrition labelling could help consumers to make healthy choices, and many different formats of nutrition labels, varying in design and complexity, are currently being used [9]. Guidelines for Daily Amounts (GDA labelling) is one example and gives consumers standards against which they can evaluate the number of calories that a food or drink serving provides [10]. In the UK, GDA labelling was introduced by many manufacturers and retailers in 1998, whereas in continental Europe, GDA's are gradually gaining acceptance [11]. Other labelling formats that have been implemented internationally are for instance the Multiple Traffic Light system, the Heart Symbol, and the Choices logo [9].

Portion size labelling could both be a promising and feasible intervention to help consumers to select appropriate portion sizes [12-14]. Especially in Europe, portion size labelling is currently not a widespread practice, and a standard format does not yet exist. However, a pilot study on the most effective format for portion size labelling indicated that providing consumers with a reference portion size was the most promising format [15].

All in all, portion size information combined with caloric GDA labelling may help consumers choose appropriate portion sizes and moderate the effects of portion distortion in a complex food environment that provides several and large portion sizes.

The few experimental studies that have explored the effectiveness of portion size labelling on consumption provide inconclusive results [16-18]. Also, previous studies have shown that once food is served, people find it difficult to regulate their intake [19]. It is therefore important to assess the impact of labelling both on portion size choices as well as on consumption. Furthermore, it is important to assess the impact of labelling in more realistic settings than the laboratory.

The aim of the present study was to assess the impact of portion size and caloric GDA labelling on consumers' portion size choices and consumption of regular soft drinks.

## Methods

### Brief Overview

**Figure 1:**
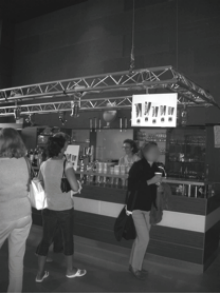
**Display material in the experimental condition**.

The study, that took place on two subsequent evenings, employed an experimental between subject design with an experimental condition with portion size and caloric GDA labelling (second evening) and a control condition (first evening). In both conditions, participants could choose between five portion sizes (200, 250, 400, 500 and 750 millilitre cups). These portion sizes were selected as being representative of the portion sizes currently available in the Netherlands. The experimental manipulation consisted of information displayed near the bar where participants ordered their drinks. In the experimental condition, portion sizes were presented on a display with both the number of portions each cup represented and the caloric GDA information (see Figure 1). Portion sizes were based on guidelines from the *Netherlands Nutrition Centre *(an institution funded by the Dutch government that provides information and education about healthy nutrition) that defines one portion of soft drink as 225 millilitres. However, as 225 millilitre cups were not the market standard, the 250-millilitre cup was designated as the reference portion. The smallest size was labelled as 0.8 portions, and the largest size was labelled as three portions. In the control condition, different portion sizes for soft drinks were displayed indicating only the amount of millilitres that each cup contained. The comprehensibility of both the portion size and the caloric GDA information was pretested with satisfying results.

### Recruitment Procedures

Participants were recruited through announcements in local newspapers, radio, and on the internet. Other recruitment methods included posting flyers in mailboxes and handing out flyers. Potential participants were told that a marketing study was conducted into consumers' attitudes towards cinemas.

Participants, unknowing of the study conditions, could choose the evening that was most convenient for them to participate. The true purpose of the study was not revealed until the conclusion of the experiment. Participants were considered eligible if they were between 21 and 65 years of age. Participants received a gift voucher worth €10, -.

### Participants

There were 101 participants in the study. After excluding participants who had not ordered a soft drink (*n *= 12), the experimental condition consisted of 48 participants and the control condition consisted of 41 participants. Overall, participants' mean age was 50.44 (12.35), 26.4% were male and 33% were overweight or obese. See Table 1 for further details.

**Table 1:** Participant characteristics

		Total sample (n = 89)	Experimental condition (n = 48)	Control condition (n = 41)
		**Mean (SD) or %**	**Mean (SD) or %**	**Mean (SD) or %**

Age		50.44 (12.35)	50.12 (12.17)	50.82 (12.71)
Sex (female)		73.6	68.8	79.5
Thirst		6.36 (2.87)	6.28 (2.73)	6.46 (3.06)
Dietary restraint		2.92 (.74)	3.00 (.68)	2.82 (.79)
External disinhibition		2.81 (.52)	2.84 (.54)	2.80 (.50)
Educational level				
	Low	8	8.3	7.7
	Moderate	50.5	45.9	56.4
	High	41.4	45.8	35.9
Weight status^1^				
	Underweight^2^	3.2	2.2	2.6
	Healthy weight^3^	63.8	68.9	53.8
	Overweight^4^	27.7	26.7	33.3
	Obese^5^	5.3	2.2	10.3
Soft drink consumption frequency				
	Never	47.1	56.3	35.9
	Seldom	32.2	27.1	38.5
	Sometimes	13.8	12.5	15.4
	Often	5.7	2.1	10.3
	Very often	1.1	2.1	0
Habitually drinks regular soft drink (when drinking soft drink)		55.3	54.3	56.4
Inferred that study was about soft drink consumption and health.		13.4	15.6	10.8
Had seen display		59.8	68.8	48.7

^1^In the Netherlands, 35% of the population are considered to be overweight and 11% are obese

^2 ^BMI < 18.50

^3 ^BMI 18.50-24.99

^4 ^BMI 25.00-29.99

^5 ^BMI ≥ 30.00

Note: No significant differences were found with respect to age, sex, BMI, dietary restraint, external disinhibition, thirst, and educational level between participants in the experimental and in the control condition.

### Study Procedures and Data Collection

A cinema was chosen as the location for the study because it is a setting in which a diverse range of people can be found. Upon arrival at the cinema, participants received the first questionnaire and were assigned a unique number and asked to write it on each questionnaire.

Participants self-completed the first questionnaire in the lobby, before the beginning of the film. This questionnaire consisted of spurious questions about the participants' previous cinematic experiences and mood. In addition, this questionnaire contained one control question measuring thirst using a visual analogue scale ranging from 0 (not at all thirsty) to 10 (very thirsty). Subsequently, participants were invited to the bar for a free regular soft drink. After participants received their drinks, they were invited to watch the film.

When the movie was finished, participants were asked to fill out the second questionnaire. The second questionnaire consisted of items that were to be used as control variables in the data analyses. The questionnaire began by asking participants what they thought was the true purpose of the study. Subsequently, they were asked a number of questions regarding their soft drink consumption (i.e. general consumption frequency, and whether they made a habit of drinking diet or regular soft drinks).

Additionally, participants were asked if they had seen the displays situated above the bar. A number of 5-point Likert items regarding the participants' opinions of the display material and their self-reported impact of the labelling were also included. To measure the participants' self-reported impact of the labelling, they were asked to rate on a 5-point Likert scale whether labelling had affected their portion size choices and soft drink consumption. Furthermore, participants were asked whether labelling had made them aware of appropriate soft drink portion sizes

Additionally, the dietary restraint and external disinhibition scales derived from the Dutch Eating Behaviour Questionnaire (DEBQ, [20]) were included in the second questionnaire. Dietary restraint (i.e. the deliberate restriction of energy intake with the intent to decrease or maintain weight [21]) was measured with a scale consisting of ten 5-point Likert items (e.g. '*Do you try to eat only a little when you want to eat a lot?'*) with α = .91. External disinhibition (i.e. overeating in response to external food-related cues such as sight and smell of attractive food [22]) was measured with ten 5-point Likert items (e.g. '*If food smells yummy, do you eat a lot of it?'*) with α = .83. The questionnaire also contained questions on gender, age, height, and body weight.

When the participants had completed the second questionnaire, they were asked to mark their participant number on their cup, and to hand in their cups and questionnaires to the research assistants. If soft drink remained in the cups, this amount was weighed afterwards. This study was approved by the VU Medical Centre's Institutional Review Board. Written informed consent was obtained from all subjects.

### Data analysis

Logistic regression and chi square analyses were run in order to assess the impact of labelling on participants' portion size choices. Since we considered it relevant to assess 1) whether labelling had an effect on selecting reference portion sizes of soft drinks, and 2) whether labelling had an effect on selecting one of the two largest soft drink sizes, the data were dichotomized and coded in two different ways. First, the portion size choices were dichotomized in order to assess whether labelling *stimulated *participants to choose the reference portion size or smaller (i.e. 250 or 200 millilitres). Therefore, participants' portion size choices were either coded as the reference portion size or smaller, or as being larger than the reference portion size. Second, portion size choices were dichotomized in order to assess the effect of labelling on *discouraging *participants from choosing one of the two largest portion sizes (i.e. 500 or 750 millilitres). Data were either dichotomized as choosing one of the two largest portion sizes, or not choosing one of the two largest portion sizes.

To assess the impact of portion size and caloric GDA labelling on soft drink consumption and to assess the self-reported impact of labelling, General Linear Model procedures were used. The dependent variable was either the amount of soft drink consumed, or the self-reported impact of labelling on 1) size choice, 2) soft drink consumption or 3) portion size awareness. Because we randomized the study conditions instead of the individual participants, we could not rule out differences in background characteristics that are likely to be related to choice and consumption behaviour of soft drinks. Therefore, both the logistic analyses and the General Linear Models were adjusted for these variables (i.e. age, gender, BMI, external disinhibition, dietary restraint, thirst, and a preference for diet versus regular soft drinks).

## Results

On the whole, 59.8% of the participants had noticed the displays (68.8% in the experimental and 48.7% in the control condition, *χ*^2 ^(1) = 3.59, *p *= .06).

### Impact of Labelling on Choice and Consumption Behaviour

**Figure 2:**
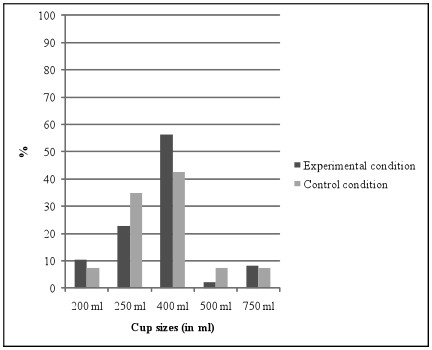
**Cup size choices (in %) in both study conditions^1^**. ^1^*χ*^2 ^(4) = 3.58, *p *= .47.

Overall, 37.5% chose the reference amount or smaller. A chi square analysis did not show a significant difference between both conditions, see Figure 2.

The logistic regression analyses indicated that portion size labelling did not increase the likelihood of choosing the reference portion size or smaller (*OR *= .75, *p *= .61, CI: .25 - 2.25). Furthermore, portion size labelling did not dissuade participants to choose one of the two largest portion sizes (*OR *= .51, *p *= .36, CI: .12 - 2.15).

Finally, no significant effects of labelling were found on soft drink consumption (experimental condition: *Mean *= 376.30, *SD *= 125.40, control condition: *Mean *= 382.14 *SD *= 147.60), *F *(1, 71) = .39, *p *= .50.

### Self-reported Impact of Labelling

With respect to the participants' self-reported impact of labelling, results showed no differences between both conditions on portion size choices, *F *(1, 46) = 2.31, *p *= .14. However, a significant interaction effect was found between labelling and gender, *F *(1, 46) = 6.66, *p *= .01. Specifically, for women the self-reported impact on choice behaviour was slightly higher in the experimental condition (*Mean *= 2.76, *SD *= 1.48) than in the control condition (*Mean *= 2.20, *SD *= 1.58). Whereas, for men the self-reported impact was lower in the experimental condition (*Mean *= 1.50, *SD *= .71) compared to the control condition (*Mean *= 3.20, *SD *= 1.48). Finally, no significant results were found for the self-reported impact of labelling on consumption *F *(1, 47) = .15, *p *= .70 or on portion size awareness, F (1, 47) = .17, *p *= .68.

## Discussion

This study was one of the first experimental studies that are known to us, that assessed the impact of portion size and caloric GDA labelling on consumers' regular soft drink portion size choices, their intake of soft drinks, and their self-reported awareness of portion sizes. The study results did not demonstrate significant effects of portion size labelling on increasing the likelihood of selecting one of the reference sizes or decreasing the likelihood of selecting the largest sizes. With respect to the latter however, it is relevant to note that the OR of selecting one of the largest sizes was lower in the experimental condition than in the control condition. A lack of power might explain that this result was not significant. Therefore, we conclude that portion size labelling did not have an effect on selecting reference portion sizes of soft drink, and that further research is needed to assess the impact of labelling on selecting large portion sizes.

With respect to the self-reported impact of portion size labelling on portion size choices, it seemed that labelling had a neutral effect on women, but a detrimental impact on men. Although this gender difference was not found for participants' actual consumption, this finding is partly in line with other studies showing that women generally attach greater importance to healthy eating than men [23] and report more health information seeking behaviour [24]. It is therefore recommended to further study gender differences in consumers' responses to labelling.

An important factor that might explain that that we found no effect for GDA labelling is that a large majority of the participants indicated that they never or seldomly drank regular soft drinks. Consequently, this could make portion size and caloric GDA labelling less relevant for them. In order to assess whether labelling was more effective among participants who reported drinking soft drinks regularly, the logistic analyses were also run among this subgroup of participants. Due to a lack of power these results could not be tested for significance, but the OR's did not indicate that portion size labelling had a beneficial impact on portion size choices (results not shown).

In this study we were interested in the effect of portion size labelling on portion size choices, as opposed to the replacement of regular products by diet products. Diet soft drinks were therefore unavailable and, as a result, participants who only drank diet soft drinks might have refused the free regular soft drink. Another consequence is that we could not test the potential effect of portion size labelling on the selection of diet soft drinks instead of regular soft drinks.

In addition, participants did not have to purchase their drinks, obviating the cost of the drink from affecting portion size choice. It is unclear how pricing would affect the impact of labelling. On the one hand, free soft drinks might have stimulated participants to select larger portion sizes than they would normally have if they had to pay. On the other hand, point of purchase settings employ value size pricing to stimulate consumers to choose large portion sizes too. Nevertheless, it would be interesting to assess the impact of portion size and GDA labelling combined with proportional pricing (i.e. eliminating beneficial pricing for large portion sizes by keeping the price per millilitre consistent).

Also, about 40% of the participants in both conditions had not noticed the displays. We chose to include all participants in the analyses, regardless of whether they had seen the displays. The reason for this was that the results from these analyses would be more generalizable to real world settings in which people often oversee nutrition labels. It is nevertheless worth mentioning that when the logistic regression analyses were run solely on participants who had seen the displays, comparable OR's were found (results not shown).

Another issue is that with respect to the participants' BMI, in this study we had to rely on self-reported data that might have suffered from a social desirability bias and under-reporting. We expect that the amount of underreporting was approximately the same for both conditions, but random measurement errors resulting from the self-reported data might still have caused some residual confounding. Last, some researchers have suggested that multiple exposures (i.e. seeing the labels more often) may be required in order for labelling to become effective [25].

Further research on portion size and caloric GDA labelling among a larger number of participants is necessary to draw more definitive conclusions. It is suggested to conduct studies that provide participants with multiple exposures to labelling and studies in which labelling is combined with pricing strategies. Future studies might benefit from more objective methods to define the participants' BMI. Last, it is recommended to gain more insight into gender differences related to labelling.

## Conclusions

Portion size and caloric GDA labelling were found to have no effect on regular soft drink portion size choices and intake. Further research with multiple exposures combined with pricing strategies among a larger number of people who have a habit of drinking regular soft drinks is recommended.

## Competing interests

The authors declare that they have no competing interests.

## Authors' contributions

WMV: data acquisition; interpretation of data; drafting the manuscript. IHMS: conception; design; critical revision of the manuscript for important intellectual content. FHL: data acquisition; critical revision of the manuscript for important intellectual content. AEB: design; critical revision of the manuscript for important intellectual content. MdB: analysis interpretation of data; critical revision of the manuscript for important intellectual content. JCS: critical revision of the manuscript for important intellectual content. All authors read and approved the final manuscript.

## Pre-publication history

The pre-publication history for this paper can be accessed here:

http://www.biomedcentral.com/1471-2458/11/438/prepub
